# Preferences for health-related quality of life: do they vary by age? A systematic literature review on the EQ-5D measure

**DOI:** 10.1007/s10198-025-01766-7

**Published:** 2025-03-25

**Authors:** Alhanouf Alabbad, Madeleine Cochrane, Paul Mark Mitchell

**Affiliations:** 1 National Pharmacovigilance Center, Saudi Food and Drug Authority, 3292 Riyadh, Saudi Arabia; 2 Health Economics and Health Policy, Population Health Sciences, Bristol Medical School, University of Bristol, Bristol BS8 1NU, UK

**Keywords:** EQ-5D Utility Values, Age-Utility Relationship, Healthcare resource allocation, Health Equity, I1

## Abstract

**Introduction:**

Cost-utility analysis (CUA) is a commonly used method in Health technology assessment (HTA) that utilises generic metrics such as quality-adjusted life years (QALYs). QALY is a measure derived from individuals’ preferences for different health states, with these preferences represented as utility values. However, utility values may differ by age, raising equity concerns in healthcare allocation. Given the globally ageing demographic, understanding the age-utility relationship becomes essential.

**Objectives:**

This systematic review aimed to explore the impact of age on utility values derived from the EQ-5D, a widely used instrument in CUA that contributes to calculating QALYs by assessing five dimensions of health: mobility, self-care, usual activities, pain/discomfort, and anxiety/depression.

**Methods:**

Our search used the comprehensive pearl growing approach and database searching. We included studies that analysed the effect of age on EQ-5D utility values in the general population. We excluded qualitative, non-English, and non-EQ-5D instrument studies. Quality was appraised using the Joanna Briggs Institute tool, and a narrative synthesis was used.

**Results:**

Of the 28 studies reviewed, primarily from Europe and the Americas, the average age of participants ranged from 34.1 and 47.7 years. Around 46% (n = 13) associated older age with lower utility values; 28% (n = 8) with higher utility; and 25% (n = 7) found no consistent relationship between age and utility.

**Discussion:**

Age was identified as a critical factor affecting EQ-5D-derived utility values, with implications for the equitable distribution of healthcare resources. These findings corroborate previous research on utility measurement across different instruments, highlighting the ethical and policy issues due to age-related utility differences.

## Background

Health technology assessment (HTA) is fundamental to evidence-based decision-making, with cost-utility analysis (CUA) being the most commonly recommended method for economic evaluation [1]. CUA evaluates interventions based on their impact on the quality and quantity of life using generic units such as quality-adjusted life years (QALYs) [2]. QALYs combine the extra years an intervention provides with the health-related quality of life (HRQOL) during those years [2]. To calculate QALYs, health states are valued on a scale where 1 represents full health, 0 implies a health state comparable to being dead, and negative values indicate states considered worse than being dead (WTD) [3]. These values, termed “utility”, reflect the strength of preference individuals have for different health states [4]. Values closer to 1 indicate health states are valued higher, while lower values indicate a lesser desirability for that health state. The utility that individuals place on various health states plays an important role in resource allocation decisions [5].

Health states can be valued directly using methods like time trade-off (TTO) and standard gamble (SG) or indirectly with generic multi-attribute utility instruments (MAUIs) like EQ-5D, SF-6D, and Health Utility Index (HUI) [4, 6]. MAUIs encompass a range of health domains and health states that patients use to characterise their health [7]. For instance, EQ-5D, the most popular MAUI, includes five dimensions: mobility, self-care, usual activity, pain/discomfort, and anxiety/depression [8]. The EQ-5D-3L has three severity levels for each dimension (no, moderate, and severe problems), while the EQ-5D-5L version has five levels (no problems, slight problems, moderate problems, severe problems and extreme problems) [8]. After completion, the questionnaire generates a score using algorithms based on utility values for the corresponding health state [6].

TTO and SG are the primary valuation techniques to estimate utility values for MAUIs [7]. The TTO method requires respondents to select the number of years spent in perfect health equivalent to a longer duration spent in sub-optimal health [9]. The lead-time TTO (LT-TTO) is used for valuing WTD health states [8]. TTO techniques are often guided by standardised protocols such as the EuroQol Valuation Technology (EQ-VT) protocol and the Measurement and Valuation of Health (MVH) study protocol for EQ-5D health states [8]. The SG method assesses the risk level, usually as a death probability, that someone would accept to avoid a particular health state [7]. Other methods, like the visual analogue scale (VAS) and discrete choice experiments (DCE), are currently less common in QALY valuation [7].

Most MAUIs, following economic evaluation guidelines, obtain utility values from the general public [10, 11]. This is consistent with the extra-welfarist perspective, which emphasizes the role of health services in enhancing societal health, using the general public’s views as a standard for evaluating health outcomes [12, 13]. However, relying on public preferences for measuring health benefits can reveal variations across different demographic groups, particularly in terms of age [7, 14]. The assumption that perceptions of quality of life remain constant across a lifespan might over-look the evolving needs and priorities as people age [15]. If significant differences in health preferences exist across age groups, this suggests that the same health intervention may offer varying benefits to different age groups [15]. Consequently, with such potential variations, the appropriateness of using standard utility scales for treatment decisions comes into question.

In the late 1970s, Sackett and Torrance became the first researchers to evaluate the general public’s perception of the utility of health states, identifying a significant correlation between age and utility in six out of fifteen health states they studied [16]. Decades later, despite the widespread application of Multi-Attribute Utility Instruments (MAUIs) and the existence of country-specific utility values derived from general populations, there is still limited understanding of how age may shape these values. This is particularly relevant in the context of a globally aging population and the drive towards equitable healthcare resource distribution. To our knowledge, no systematic review has been published that explores the effect of age on utility values derived from MAUIs. Given the prevalent use of EQ-5D among other instruments and its frequent recommendation in various guidelines, it becomes the focus of this review [1, 6]. Our objectives are:

To systematically review the existing literature on age’s impact on utility values, estimated for EQ-5D measures within the general population.

To investigate how age influences the estimated utility values of EQ-5D measures across different quality-of-life dimensions.

## Methods

### Search strategy

We adopted a dual-method search approach for our review. Due to the variability in keywords indexing our topic of interest, we initially employed the comprehensive pearl growing (CPG) method. This method is particularly beneficial for topics that span multiple domains or have varied terminology [17]. The CPG starts with a specific article or group of articles closely aligned with the research objective, commonly termed “key pearls” [18]. From these, a “first wave of pearls” is identified, consisting of articles that cited the initial key pearls [19]. This iterative process continues until no further relevant articles are found in subsequent waves. After completing the CPG search, we proceeded with a traditional database search to ensure comprehensive literature coverage. We also checked the reference lists of all included papers.

### Selection of key pearls

Our preliminary search identified three key pearl articles from the Measurement and Valuation of Health (MVH) study conducted by researchers at the University of York in the UK in 1994, which aimed to generate utility values for the EQ-5D from the public [20]. These articles examined the impact of age on the elicited utility values [21–23]. Given the MVH’s distinction as the first TTO-based EQ-5D valuation study, it is unlikely that any significant prior literature was overlooked. We initiated the citation search via Web of Science, a comprehensive platform covering databases like Medline, which offers indexed and searchable references across multiple disciplines [24]. Papers published between 1994 and July 2023 were included in the search.

### Database searching

We conducted a search spanning 1994 to July 2023 using the OVID platform, which covered the PsycINFO, Pub-Med, and Embase databases, selected for their relevance and scope. Our search terms focused on the general population or “older” individuals within the general population. We primarily examined the EQ-5D instrument, considering utility values as the main outcome. Searches were restricted to English publications. For a complete list of search terms and the PICO framework, see Appendix 1(Online resource 1).

### Study selection

Studies were included if they:

1- Investigated the effect of age on EQ-5D utility values and
2- Derived utility values from the general population.

Studies that focused on the influence of age on health state utility were labelled as “primary studies”. Studies that had different main objectives but still considered age’s influence in their analyses were labelled as “secondary studies”. Both primary and secondary studies were included in our selection. Studies that reported outcomes related to utility values, like the number of years traded off or the number of states deemed WTD, were also included.

Feasibility or explorative studies were not excluded. We excluded qualitative studies, non-EQ-5D measurements, and studies focusing solely on specific patient groups to maintain broad relevance. The initial screening was done based on titles and abstracts. Subsequently, relevant studies underwent full-text review. One reviewer managed the selection, another reviewed 10% of the sample, and disagreements were resolved with a third reviewer.

### Risk of bias

We used the Joanna Briggs Institute (JBI) critical appraisal tool for analytical cross-sectional studies to assess the risk of bias in the included studies [25]. This tool evaluates methodological quality by assessing inclusion criteria, setting descriptions, measurement methods, handling confounding variables, and statistical analysis. The tool includes eight questions that can be answered with “yes”, “no”, “unclear”, or “not applicable”. Item 4, given its clinical focus, was considered irrelevant to all the studies. A single reviewer applied the checklist independently.

### Data extraction

One reviewer extracted data into a spreadsheet. The extracted data covered bibliographic details (authors, publication date, country), objectives, demographics (age range, mean, and standard deviation), EQ-5D version, methods (design, sample size, sampling and data collection, adherence to the EQ-VT or the MVH protocols, existence of a protocol for WTD states, number of states valued, and statistical analysis), and outcome data.

### Analysis

We adopted a structured narrative synthesis approach to address the variety of outcomes reported in the reviewed studies. Our analysis highlighted age-related patterns in utility values obtained from the general population. In this context, utility values on the EQ-5D questionnaire, ranging from 0 to 1, serve as a measure of the perceived quality of different health states, with higher utility values indicating more positive perceptions. We determined that for each health state, an increase in age coupled with a higher utility value, a lower disutility value, or fewer years traded off signaled a positive association between utility and age. This suggests that as individuals age, they tend to view health states more favorably, or at least, less negatively. Conversely, an increase in age accompanied by a lower utility value, a higher disutility value, or more years traded off represented a negative association, indicating a decline in the perceived quality of these states with advancing age. Our investigation further explored whether age’s impact on perceptions was more pronounced in certain health domains or severity levels. To understand these patterns, relationships within and between studies were explored, with an emphasis on comparing primary and secondary studies.

## Results

### Study Selection

**Fig. 1:**
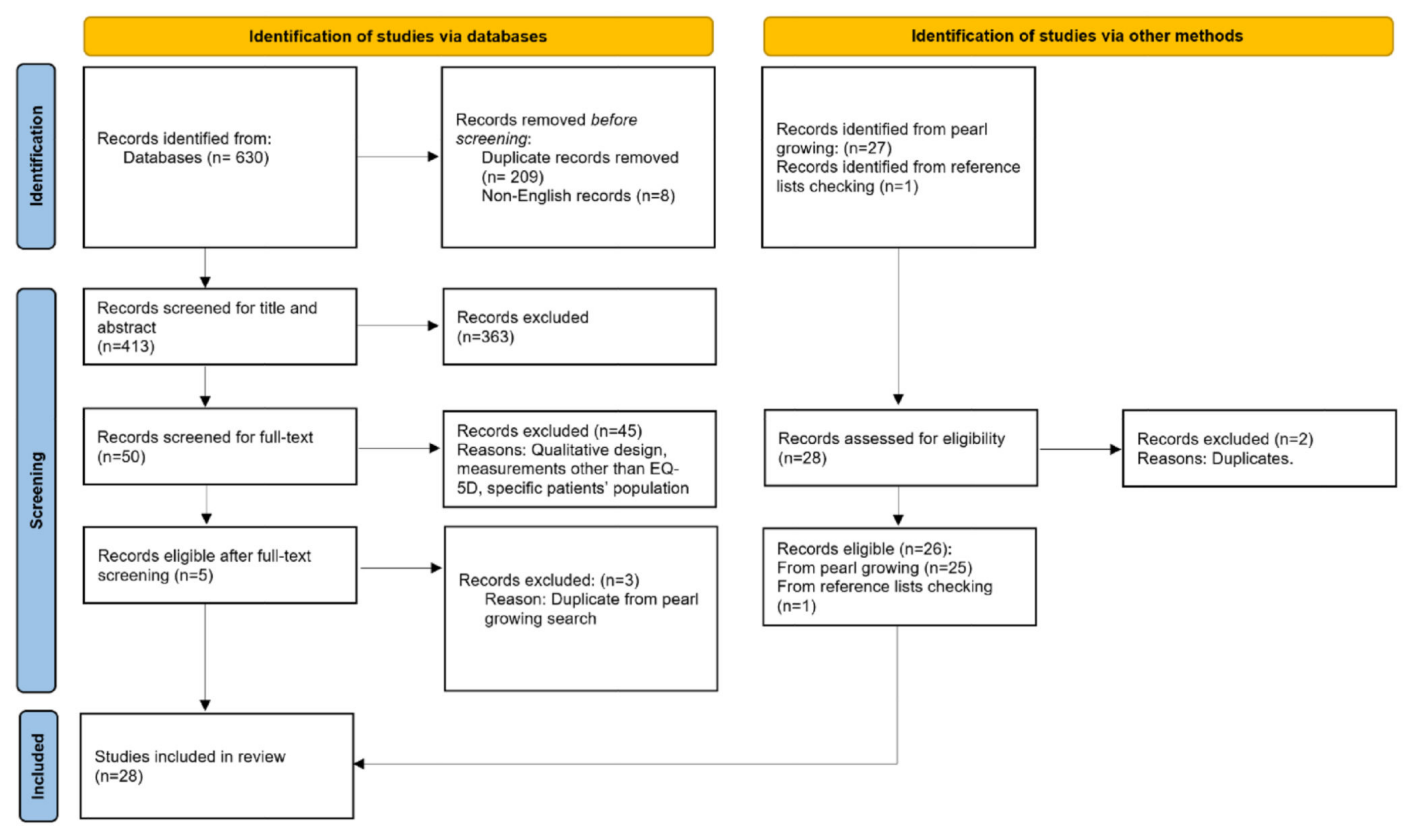
PRISMA flow diagram representing the study selection process [51]

**Fig. 2:**
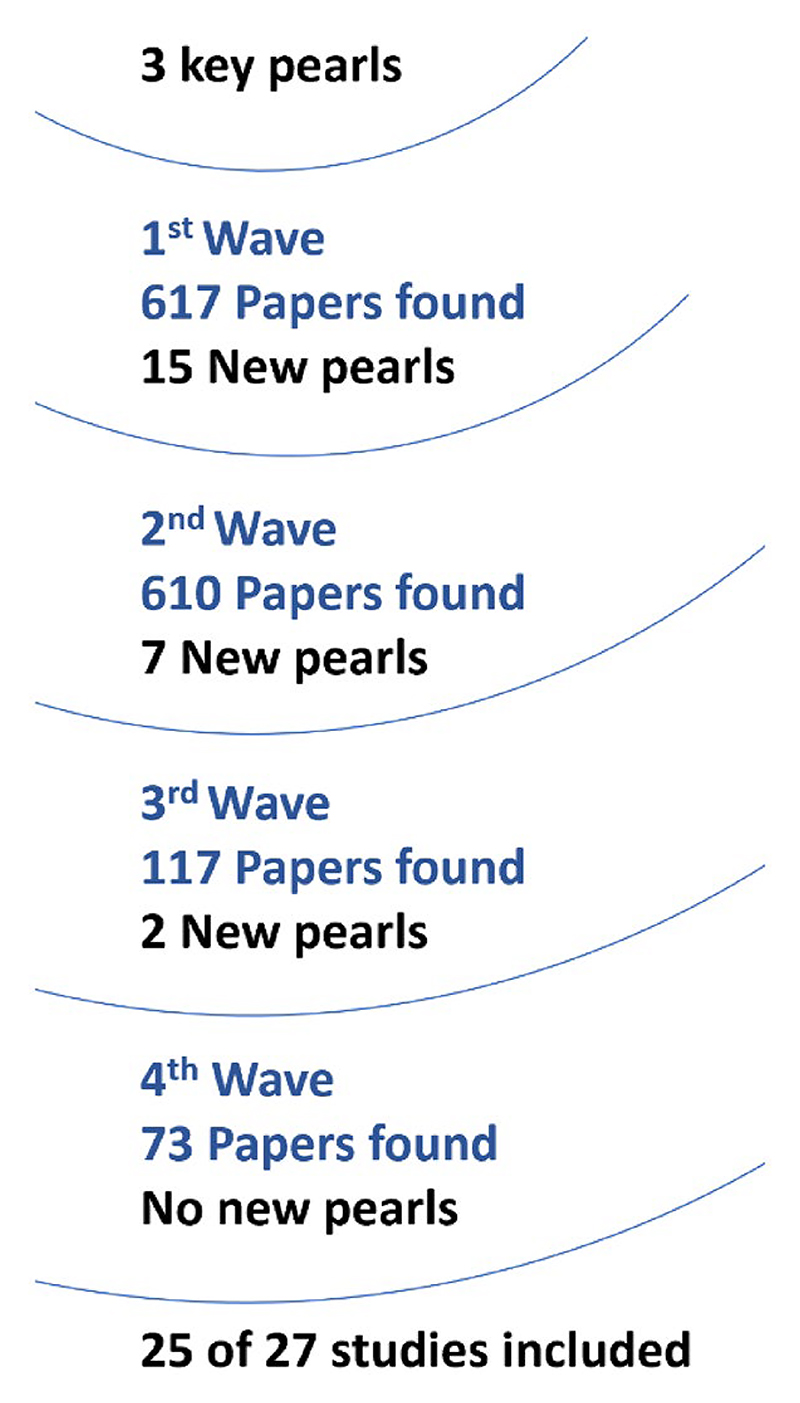
Summary statistics of initial CPG search results

As shown in Fig. 1, our dual-method search yielded 28 studies [21–23, 26–50]. The CPG method identified 25 studies across the four waves of literature searches (see Fig. 2) [21–23, 27–33, 35–42, 44–50]. One additional study was found by checking reference lists [26]. Our database search retrieved 630 records; after deduplication and initial screening, 50 were considered relevant. Out of these, five matched our eligibility criteria. However, three of these five over-lapped with the CPG method, leaving two unique records [34, 43]. When combining all search methods, we identified 28 studies.

### Characteristics of studies

Table 1 displays study characteristics by country and year. The majority (71%, N = 20) were from Europe [21–23, 26–28, 30, 32–37, 41–43, 45–47, 49], while others were from the Americas (US [29, 31], Canada [40], Brazil [38, 48]), Asia (China [39, 44]) and Africa (Egypt [50]). Five studies used the same UK MVH data set [21–23, 43, 45], with two studies using US data alongside the UK MVH data set [29, 34]. Two Brazilian studies [38, 48] and two Dutch studies [36, 41] drew from the same data sets in each country, respectively. One study drew from 28 different value sets from across the world [47].

Eleven studies (39%) were considered primary studies, and seventeen (61%) were secondary (see Tables 2 and 3). Sample sizes ranged from 105 to 29,140, and participant ages averaged between 34.1 ± 13.5 and 47.7 ± 18.1.

### Quality assessment

Using the Joanna Briggs Institute (JBI) Quality Appraisal Tool, most studies were high-quality, but eight had concerns regarding one or two items from the JBI tool [21, 28, 30, 32, 37, 43, 44, 46]. One study had issues with three items [47]. (see Appendix 2, (Online resource 2)).

### Methodological approach and valuation methods

The majority of studies (N = 27, 96%) used the TTO method; among these, three studies combined TTO with VAS. Only one article relied solely on VAS [26].

The EQ-5D-3L was predominantly used, but some papers used the EQ-5D-5L [39, 40, 42, 46, 47, 49, 50] Additionally, one study chose the EQ-5D+, which adds a cognitive domain to the existing five domains [30]. Seventeen studies (61%) followed EQ-VT or MVH protocols. Most papers analysed the age effect on mean utility values (TTO scores). Some investigated relationships between age and metrics like the number of years traded off, WTD states [33, 35, 36, 41, 42, 49], or the difference in utility values between mild and severe states [47]. One explored multiple TTO-based indicators [39].

### Age’s influence on utility values associated with EQ-5D health states

#### Age and lower utility values

Our findings showed that older age groups tend to have more negative perceptions of EQ-5D health states. This was demonstrated by their tendency to assign lower utility values or trade off more years to avoid less desirable health states. This was evident in 46.4% of the studies examined (N = 13, five primary and eight secondary studies) [21–23, 29, 31, 34, 40, 42, 43, 45, 46, 49, 50].

Seven studies used UK MVH data set [21–23, 29, 34, 43, 45]. Dolan et al. conducted three key studies; the first showed utility values were higher up to age 40 and then became lower beyond that age, with a notable decrease post-60, especially for severe health states [22]. These observations were validated by Kharroubi et al. using a different model [43]. Dolan’s later research categorised age groups as ≥ 60 and < 60 and found higher disutility for the older group. However, this effect softened when scores were adjusted, considering that older people might find WTD states implausible [21]. In the third study, Dolan demonstrated that 45-year-olds rated utility around 0.07 units higher than 70-year-olds on a 1 to 0 scale [23]. Further sub-group analysis by Cubi-Molla et al. indicated that respondents aged 68 + valued certain health states up to 0.40 lower than younger participants [45].

This trend was supported by two other EQ-5D-5L studies from the UK [42, 46] and one study from the US [31]. A synthesis of UK MVH and US data suggested a slightly stronger age effect in the UK [29, 34]. Findings from Canada and Egypt also identified age as a determinant of utility values, with seniors (> 60 years) consistently scoring lower values [40, 50]. In Norway, older cohorts traded off more years to avoid undesirable health states than younger ones, though the trend was less pronounced for cohabiting individuals [49].

#### Age and higher utility values

Eight papers (28.5%), including three primary and five secondary studies, showed a positive link between advancing age and utility values. In these studies, older individuals attributed higher utility values to EQ-5D health states or showed less willingness to trade life years to avoid poor health states (see Tables 2 and 3) [26, 32, 33, 36, 38, 41, 44, 48]. This subset of research suggests that some older adults may perceive certain health states more positively than their younger counterparts.

Three Dutch studies found older participants less willing to trade off years [33, 36, 41]. Analysis from the Brazilian valuation dataset found that each added year of age was linked to a 0.0019 increase in utility values and for the poorest health state, there was a 0.1 value difference on a 1-to-0 scale between those aged 60–64 and 18–19 [38, 48]. Similar findings were reported in two other studies from Poland and China, where older participants were found to assign either higher utility or lower disutility values compared to younger age groups [32, 44].

Another study, relying on VAS and sourcing participants from one medical centre, noted a minor age-positive effect: older participants assigned higher utility values to better health states [26].

#### Inconsistent or negligible relationship between age and utility

The relationship between age and utility was either negligible or inconsistent in seven papers (25%) (see Tables 2 and 3) [27, 28, 30, 35, 37, 39, 47]. One study found that the influence of age on utility depended on the valuation method used [35], while the others found no significant relationship between age and utility [27, 28, 30, 37, 39, 47].

### Impact of age on quality-of-life dimensions

Two studies from the MVH study explored age-related differences in utility values across quality-of-life dimensions [21, 45]. Dolan et al. found that both age groups (≥ 60 and < 60) were concerned about pain, but the older group found difficulties with washing or dressing more challenging than younger individuals [21]. By introducing further subgroup analysis to six age groups, Cubi-Molla et al. identified that the oldest adults (aged > 68) displayed distinct utility values for states marked by level 3 in mobility or levels 2 and 3 in self-care [45].

## Discussion

### Main findings

This review investigated variations in utility values among different age groups using the EQ-5D instrument. Across the majority of studies, a clear trend was observed: age influences utility values. Older individuals assigned different values to health states than younger individuals. However, the direction and magnitude of this effect varied among studies. This conclusion is supported by the quantitative trends observed in our data synthesis and the qualitative insights from studies exploring the underlying reasons behind these differences. For example, among the A. Alabbad et al. five EQ-5D dimensions, older individuals placed higher importance on self-care and mobility, which might reflect a shift in priorities towards maintaining independence and functional capabilities as people age.

When specifically examining primary studies, which directly investigated the influence of age on utility, we encountered mixed results regarding the direction of age’s effect. Some studies reported that older age was associated with lower utility values, while others identified higher values or found the effect to be negligible. Conversely, secondary studies, which did not primarily focus on our research question but still considered the influence of age, similarly documented a range of effects, further illustrating the nuanced relationship between age and utility valuations.

Our analysis highlighted a wide range of methodologies used across the studies, encompassing various valuation methods (like time trade-off (TTO) with a 10-year horizon, TTO adjusted for life expectancy, and visual analogue scales (VAS)), the outcomes measured, questionnaire methodologies, and compliance with specific valuation protocols. This wide range of methods, together with potential differences in cultural beliefs among countries, variations in health systems, and the representation of various age groups, likely plays a significant role in the variability of findings observed.

Broadly, our findings resonate with existing literature. Research on other generic MAUIs, such as the SF-36 and HUI, consistently indicated age’s influence on utility values, especially in more severe states [52–54]. Data from multiple studies assessing age’s effect on diverse condition-specific health states suggested older participants generally showed lower utility values than younger people [55]. Relevant to our study, a systematic review of patient heterogeneity in economic evaluations identified age as a potential factor influencing health state utility [14]. Sackett and Torrance, in their pioneering effort to measure societal utility values across multiple disorders, noted a subtle age effect for some, but not all, valued scenarios [16]. This observation mirrors our results, as we documented age’s impact varying across severity levels and quality of life dimensions.

When analysing specific dimensions, we found that older individuals notably favoured mobility and self-care. This is consistent with qualitative and quantitative research highlighting older adults’ emphasis on functional ability [56, 57].

The influence of age on utility is multifaceted. On the one hand, age might inherently shape health preferences. Dolan et al. suggested this could arise from older people’s desire not to burden their families, citing qualitative data from the MVH study [58]. On the other hand, the valuation methods may have an artefact effect. The standard valuation protocols employ a 10-year horizon for the TTO exercise [8]. However, the perception of a decade might differ significantly based on the respondent’s age. For the younger cohort, 10 years could seem short, while for older adults, it might appear a generous time, and they might be more willing to sacrifice years they did not expect to reach [22]. Nooten et al.’s research demonstrated that, while there was no direct correlation between age and willingness to trade off years, there was a link between one’s subjective life expectancy (SLE), with those expecting a shorter life being more willing to trade off years [33]. Similarly, Bot et al. found that when using two TTO variations, a 10 year TTO and a life expectancy TTO-LE, the average TTO-LE valuations were consistently lower than those derived using the 10 year TTO [30]. Another point to consider is older individuals’ scepticism towards WTD health states. Data from the MVH indicated that only 10% of individuals below 60 found such states implausible, compared to 50% of older participants [21]. However, adjusting for this variable did not completely eliminate the age effect.

### Strengths and limitations of evidence

This review is based on 28 studies, 17 of which adhered to established protocols like EQ-VT or MVH. These studies were often distinguished by their performance against the JBI quality tool, their large samples, and their diverse age demographics, which supported the credibility of their results. Conversely, studies not conforming to these protocols frequently encountered challenges in quality assessment and exhibited limitations, including smaller sample sizes or skewed age representation. Such factors could compromise the general applicability of their conclusions. Furthermore, the frequent use of certain datasets, notably the MVH study, across various studies ensures a level of methodological consistency, supporting the strength of their findings. However, this also poses a limitation to the diversity of the data, potentially limiting its applicability across different populations and health systems.

### Strengths and limitations

To our knowledge, this is the first review addressing the age-utility relationship reported in studies using the EQ-5D instrument. Our dual search strategy increased the likelihood that all relevant literature had been included. However, only two studies addressed the influence of age on health dimensions, suggesting alternative search strategies might offer a more comprehensive understanding of this aspect.

This review is not without its limitations. Findings were limited by what variables the study authors included in their studies (see Appendix 3), this means the results on age’s impact on utility might be influenced by any omitted variables that interplay with age. Additionally, although our searching and selection processes involved two reviewers, the bias assessment, data extraction, and evidence rating were managed by a single reviewer. Lastly, it is important to recognize that our analysis was exclusively based on the EQ-5D instrument. Including other measures, such as the ICECAP, might offer different insights into the age-utility relationship.

### Policy implications and future research

Our review suggests age as a determinant of utility values. However, the variations in the strength and nature of this relationship which we observed in our findings, emphasises the need for research tailored to understand the age-utility dynamic. If systematic variations in utility values among different age sub-groups are confirmed, they could pose ethical and policy dilemmas, particularly if age-based utility values render certain treatments cost-effective for one age group but not for another. The use and potential implications of different subgroup valuation sets have been explored previously [45, 59]. Such outcomes would inevitably raise concerns about the equity of healthcare resource distribution. Future reviews could be strengthened by adopting a more granular approach to data synthesis. This includes conducting meta-analyses where possible, to quantitatively aggregate utility values across studies, and exploring how specific methodological aspects, such as the statistical analysis undertaken and choice of control variables, might influence the observed age effect.

## Conclusion

By systematically reviewing the relevant literature, we found that within the EQ-5D framework, older people tend to value the same health states differently from younger people. Nevertheless, our findings should be contextualised by considering the limitations inherent in the studies reviewed and the potential constraints of our review methodology.

## Supplementary Material

Supplementary file1

Supplementary file2

Supplementary file3

## Figures and Tables

**Table 1:** Characteristics of the selected studies (n = 28)

Lead Author/Year	Country	Study Population	Sampling Technique	Sample Size	Format	Total States: Per Respondent	Valuation Method	Age	Age Mean/SD	Age Representatives	EQ-VT/MVH Protocol	WTH Protocol	The Direction Of Age Effect
Dolan et al., 1996 [22]	UK	GP	Quota	3337^a^	FI	43:13	TTO 10	18 - 60+	47.7 ± 18.3	✓	✓	✓	
Dolan, P, 2000 [21]	UK	GP	Quota	2997^a^	FI	42:12	TTO 10	18 -60+	47.7 + 18.1	✓	✓	✓	
Dolan, P. et al., 2002 [23]	UK	GP	Quota	2997^a^	FI	42:12	TTO 10	18 -60+	47.7 + 18.1	✓	✓	✓	
Barry, L. et al., 2018 [42]	UK	GP	Random	160	FI	84:10	TTO 10	18 - 60+	NR	NR	✓	✓	
Kharroubi, S.A., et al., 2018 [43]	UK	GP	Quota	2997^a^	FI	42:13	TTO 10	18 -60+	47.7 + 18.1	✓	✓	✓	
Cubi-Molla, P. et al., 2019 [45]	UK	GP	Quota	2997^a^	FI	42:12	TTO 10	18 -60+	47.7 + 18.1	✓	✓	✓	
Spencer et al., 2019 [46]	UK	GP	Online panel	1339	OS	8:4	TTO 20	18 - 70	45+14	X	X	X	
Shaw et al, 2007 [31]	US	GP	Probabilistic	3773^b^	FI	42:12	TTO 10	18 - 99.3	44.5 + 30.7	✓	✓	✓	
Johnson, J.A. et al, 2005 [29]	US/UK	GP	Quota Probabilistic	2997^a^ 3773^b^	FI	42:12	TTO 10	18 -60+	47.7 + 18.1 44.5 ± 30.7	✓	✓	✓	
Kharroubi, S.A, et al, 2010 [34]	UK/US	GP	Quota Probabilistic	2997^a^ 3773^b^	FI	42:12	TTO 10	18 -60+	47.7 + 18.1 44.5 + 30.7	✓	✓	✓	
Santos, M, et al, 2016 [38]	Brazil	GP	Probabilistic	9148^c^	FI	243:7	TTO 10	18 - 64	37.8 + 13.1	X	✓	✓	+
Santos, M. et al, 2020 [48]	Brazil	GP	Probabilistic	5774^c^	FI	243:7	TTO 10	18 - 64	37.8 + 13.1	X	✓	✓	+
Sayah, F.A. et al, 2016 [40]	Canada	GP	Quota	1209	FI	86:10	TTO 10	18 - 89	47.6 ± 17.4	✓	✓	✓	-
Al Shabasy, S. et al, 2022 [50]	Egypt	GP	Quota	972	FI	86:10	TTO 10	18 - 72	36.9 ± 12.7	X	✓	✓	-
Jakubczyk, M, 2009 [32]	Poland	PMC users	Quota	295	FI	44:23	TTO 10	18 - 86	42.8 ± 15.7	X	X	✓	+
Jin et al, 2016 [39]	China	GP	Quota	1296	FI	86:10	TTO 10	16 - 60+	42.3 ± 16.2	✓	✓	✓	/
Zhuo, L. et al, 2018 [44]	China	GP	Random	5503	FI	NR:1 2	TTO 10	15 - 97	NR	X	X	✓	+
Augestad et al, 2013 [35]	Norway	GP	Online panel	629	OS	8:8	TTO 10 LT-TTO VAS	18 - 80+	NR	X	✓	✓	+ - ?
Hansen, T.M, et al, 2022 [49]	Norway	GP	Random/quota	430	FI	84:10	TTO 10	20 - 80	43.8 + 15.9	NR	✓	✓	-
Badia, X, 1995 [26]	Spain	PMC users	Quota	600	SS	13:13	VAS	16 - 76+	NR	X	✓	NA	+
Badia et al. 1999 [27]	Spain	PMC users	Random	294	FI	43:13	TTO 10 VAS	18 - 64+	NR	✓	X	✓	/
Van Nooten et al, 2004 [28]	Netherlands	GP	Convenience	105	SS	3:3	TTO 80	NR	34.1 + 13.5	X	X	X	/
Bot et al, 2007 [30]	Netherlands	GP	NR	178	OS	6:6	TTO10 TTO-LE VAS	20 - 25 45 - 50 60 - 65	NR	X	X	X	/
Van Nooten F. E. et al, 2009 [33]	Netherlands	GP	Online panel	339	OS	3:3	TTO 10	18 - 65	42.89 ± 13.28	✓	X	X	+
Van Nooten, F.E. et al, 2015 [36]	Netherlands	GP	Online panel	1067^d^	OS	3:3	TTO 10	18 - 65	43 ± 13.64	X	X	X	+
Krol, M.etal, 2016 [37]	Netherlands	GP	NR	1446	OS	4:4	TTO 10	NR	47+15	NR	✓	X	/
Van Nooten, F. E. et al, 2017 [41]	Netherlands	GP	Online panel	1067^d^	OS	3:3	TTO 10	18 - 65	43 ± 13.64	X	X	X	+
Roudijk B et al, 2019 [47]	Netherlands	28 EQ-5D valuation studies	NA	29140	NA	NA	TTO-10	NR	NR	NR	X	NR	+ - ?

a Studies using the same UK value set;

b Studies using the same US value set;

c Studies using the same Brazilian value set;

d Studies using the same LXitch value set.

WTD: worse than dead health Mates. GP: general population, PMC-primary medical centre, FI: face-to-face interviews, SS: self-administered survey, OS: online survey, TTOIO: 10 yean time horizon, TTO 80: lifetime horizon assuming lifetime of 80 years, TTO-LE: life expectancy time horizon. I.T-TTO: lead time TTO, NR: not reported, NA: not applicable

▭ higher utility with age (+)

▭ lower utility with age (-)

▭ insignificant result (/) or mixed results (+ - ?)

**Table 2:** Summary of results from EQ-5D *primary v*aluation studies examining the impact of age on utility values and associated outcomes (n = 11)

Study ID	Objective	EQ-5D version	Age variable type	Specific outcome measure	Age impact on outcome	Impact on severity level/dimensions	Additional findings
Dolan 2000, UK [21]	To estimate EQ-5D values based on age and examine the effect of modifying values for older respondents	3L	Cat.^a^ (18–59) and 60 +	Mean disutility values (1-TTO)	Disutility values were higher for older age groups	All severity level/Self-care	Older individuals (≥ 60) viewed all levels and types of dysfunctions as worse than younger ones, especially when their scores were unmodified. Modified scores considered the possibility that older respondents might have found the WTD scenario implausible
Dolan et al., 2002, UK [23]	To explain TTO values for EQ-5D health states in terms of respondent characteristics and VAS scores	3L	Cont.^b^	Mean utility values (TTO)	Utility values decreased as age increased	NR	The relationship between age and utility values followed a quadratic trend: values increased until around 45, tapered off until 70, and then sharply dropped. On an impact scale of 1 to 0, a 45-year-old valued a health state roughly 0.075 higher than a 70-year-old
Cubi-Molla et al., 2019, UK [45]	To expand on the relationship between age and utility in the MVH data	3L	Cat.^a^ (18–27), (28–37), (38–47), (48–57), (58–67), and 68 +	Mean utility values (TTO)	Utility values were lower for older age groups	Moderate and severe states/Mobility and self-care	Older groups valued some health states as WTD, whereas younger groups perceived them as better than dead. On an impact scale from 1 to 0, the difference in utility values was 0.4
Santos et al., 2020, Brazil [48]	To explore predictors of the TTO valuation results	3L	Cont.^b^	Mean utility values (TTO)	Utility values increased as age increased	NR	None
Sayah et al., 2016, Canada [40]	To examine how demographic and health factors affect TTO valuations in the Canadian EQ-5D-5L study	5L	Cat.^a^ (18–28), (40–59), and (60–89)	Mean utility values (TTO)	Utility values were lower for older age groups	NR	Older adults (60+) scored lower utility values than younger ones, even after accounting for factors like education and gender
Al Shabasy et al., 2022, Egypt [50]	To examine how demographic and health factors affect TTO valuations in the Egyptian EQ-5D-5L study	5L	Cat.^a^ (18–30), (31–45), (46–60), and (60+)	Mean utility values (TTO)	Utility values were lower for older age groups	NR	Older adults (60+) scored lower utility values than younger ones, even after accounting for factors like education and gender
Jakubczyk 2009, Poland [32]	To identify the determinants of HRQoL, focusing on the influence of interactions within and between health states and demographics on state utility	3L	Cont.^b^	Mean disutility values (1-TTO)	Disutility values decreased as age increased	NR	As age increased, disutility (i.e., 1 – utility) decreased, implying that older people assigned higher utility values for health states
Jin et al. 2016, China [39]	To determine the cultural and demographic factors that influence health preferences in China	5L	Cont.^b^	Four indicators TTO = 1: If participants were willing to trade any years to avoid suboptimal health states. TTO>0: If participants assigned positive values. TTO = -1: If participants gave the lowest possible utility value. The TTO range	No significant relationship was found between age and all four indicators	NR	None
Badia 1995, Spain [26]	To assess the feasibility of EuroQol and examine how self-rated health and sociodemographic variables impact health state valuations	3L	Cat.^a^ ≤ 60 and >60	Mean utility values (VAS)	Utility values were higher for older age groups	Better health states	None
Bot et al., 2007, Netherlands [30]	To distinguish between the effects of respondents’ age and time preference on the valuations for hypothetical health states	EQ-5D +	Cat.^a^ (20–25), (45–50), and (60–65)	Mean utility values (TTO 10, TTO LE, VAS)	No significant differences were found between age groups	NR	Mean TTO-LE (with life expectancy) values were generally lower than TTO-10 and VAS. There were no differences in utility values between age groups across all three elicitation methods. Age groups accounted for a very small variance (0.2%) in utility values
Roudijk et al., 2019, Netherlands [47]	To assess how sociodemographic. methodology. and cultural values affect health utility differences	3L and 5L	Cont.^b^	The difference in mean utility values between mild and severe states	No significant relationship was found between age and differences in utility values	NR	EQ-5D valuation studies from 27 countries revealed a weak correlation between age and differences in utility values, both within individual countries and when aggregated across the 27 countries

a Age was treated as a categorical variable

b Age was treated as a continuous variable

*NR* not reported For full statistical results, please refer to Appendix 3, (Online resource 3)

**Table 3:** Summary of results from EQ-5D *secondary* valuation studies examining the impact of age on utility values and associated outcomes (n = 17)

Study ID	Objective	EQ-5D version	Age variable type	Specific outcome measure	Age impact on outcome	Impact on severity level/dimensions	Additional findings
Dolan et al., 1996, UK [22]	To generate an EQ-5D value set for the UK general population	3L	Cont.^b^	Mean utility values (TTO)	Utility values decreased as age increased	Moderate and severe states	There was a quadratic relationship between age and utility values. Values slowly increased from age 18 to about 40. After age 40, these valuations gradually decreased until around age 60, then sharply declined
Kharroubi et al., 2018, UK [43]	To apply a non-parametric Bayesian approach to model UKEQ-5D valuations	3L	Cont.^b^	Mean utility values (TTO)	Utility values decreased as age increased	All severity levels, but more pronounced in the severe state	Severe states showed a quadratic age relationship: valuations were less negative until ages 40–45, then became more negative, particularly in advanced ages (70+) Mild states displayed a mild inverted ”U” pattern, peaked at ages 40–60. then declined
Johnson et al. 2005, US [29]	To develop a US-based preference weighting system for EQ-5D’s health states and compare it with UK valuations	3L	Cont.^b^	Mean utility values (TTO)	Utility values decreased as age increased	NR	The US sample was younger than the UK’s. The US mean valuations for EQ-5D health states were higher than the UK’s. There was a quadratic relationship between age and utility values
Kharroubi et al., 2010, UK [34]	To introduce a non-parametric Bayesian model analysing US-UK EQ-5D~data	3L	Cont.^b^	Mean utility values (TTO)	Utility values decreased as age increased	NR	The age effect was slightly more pronounced in the UK
Barry et al., 2018, UK [42]	To explore the impact of religiosity and views on euthanasia on the propensity to assign a health state as “WTD”	5L	Cat^a^ (18–35), (36–45), (46–60), and 61 +	The probability of assigning negative values (WTD)	The probability of assigning negative values to a health state increased as age increased	NR	None
Spencer et al., 2019, UK [46]	To examine if attitudes towards life length and quality influence TTO values	5L	Cont.^b^	Mean utility values (TTO)	Utility values decreased as age increased	NR	There was a quadratic relationship between age and utility values. Values tended to increase with age, but this trend reversed at a certain point
Shaw et al., 2007, US [31]	To explore how race/ethnicity and socio-economic status influence preferences for EQ-5D health states in the U.S	3L	Cont.^b^	Mean utility values (TTO)	Utility values decreased as age increased	NR	There was a quadratic relationship between age and utility values. Values increased until middle age and then decreased after that
Santos et al., 2016, Brazil [38]	To generate an EQ-5D value set for the Brazilian general population using all 234 health states	3L	Cont.^b^	Mean utility values (TTO)	Utility values increased as age increased	Severe states	A linear association between age and utility values was evident for the poorest health state (33,333). As age increased, values increased. On an impact scale from 1 to 0. the scores for the oldest age group (60–64) were approximately 0.1 higher than those for the youngest (18–19)
Hansen et al., 2022, Norway [49]	To investigate the effect of having significant others, including children and a partner, on TTO valuations	5L	Cat.^a^ Four age quantiles (Details not provided)	Mean disutility values (1-TTO) and number of years traded off	Disutility values and the number of years individuals were willing to trade off were higher for older age groups	NR	The older quartiles, 3 and 4, showed greater disutility than the younger quartiles. This age-related increase in disutility was more pronounced for those not living with significant others
Badia et al. 1999, Spain [27]	To evaluate the feasibility. validity, and reliability of TTO and VAS methods in a Spanish sample	3L	NR	Mean utility values (TTO and VAS)	Age had a very slight effect on utility values	NR	Age slightly impacted TTO and VAS scores, affecting the values of only three states
Augestad et al., 2013, Norway [35]	To examine how attitudes towards euthanasia affect health state values elicited through TTO, LT-TTO, and VAS methods	3L	Cont.^b^	Mean utility values (TTO, LT-TTO, and VAS) and number of health states valued WTD	Mixed results. Age impact on utility values and the number of WTD states depended on the valuation technique	NR	When using TTO and VAS techniques, utility values increased as age increased. However, with the LT-TTO method, no significant relationship was observed The number of health states valued WTD increased with age using the LT-TTO method and decreased when using the VAS method
Van Nooten et al., 2009, Netherlands [33]	To investigate the influence of subjective life expectancy (SLE) on responses in a 10-year time trade-off exercise	3L	Cont.^b^	Willingness to trade off years and the number of years traded off	The number of years traded off decreased as age increased The willingness to trade off years was not affected by age	NR	While age did not affect the willingness to trade off, people with lower SLE were more willing to trade off As age and SLE increased, the number of years traded off decreased
Van Nooten et al., 2015, Netherlands [36]	To explore the impact of having a partner and children on TTO scores	3L	Cont.^b^	Number of years traded off	The number of years traded off decreased as age increased	NR	With increasing age. respondents were willing to give up fewer years. The results remained significant even after including variables related to having children and living with a partner
Van Nooten et al., 2004, Netherlands [28]	To assess how personal beliefs about the age of death and future quality of life influence TTO responses	3L	Cont.^b^	Mean utility values (TTO) and number of years traded off	No significant relationship was found between age and mean utility values	NR	A higher expected age of death corresponded to higher utility values. Individuals who expected to live beyond 80 traded fewer years than those who anticipated not reaching 80
Nooten et al., 2017, Netherlands [41]	To identify and characterise subgroups within the general public of the Netherlands based on their TTO responses	3L	Cont.^b^	Number of years traded off	The number of years traded off decreased as age increased	NR	Four sub-groups were identified based on the number of years respondents traded off (low, medium–low, medium-high, and high traders) Older individuals were more likely to be “low traders”, suggesting they were less willing to trade off life years than younger ones
Krol et al., 2016, Netherlands [37]	To explore the existence and impact of altruistic preferences on health state valuations using TTO	3L	Cont.^b^	Mean utility values (TTO)	No significant relationship between age and mean utility values	NR	Respondents who thought of being a burden to their loved ones had lower utility values, whereas those who believed their loved ones would miss them had higher utility values
Zhuo et al., 2018, China [44]	To derive a value set for the EQ-5D-3L from the general Chinese population	3L	Cat.^a^ (15–24), (25–34), (35–44), (45–54), (55–64), (65–74), and (75–97)	Mean utility values (TTO)	Utility values were higher for older age groups	NR	None

a Age was treated as a categorical variable

b Age was treated as a continuous variable *NR* not reported

For full statistical results, please refer to Appendix 3, (Online resource 3) Preferences for health-related quality of life: do they vary by age? A systematic literature…

## Data Availability

The authors confirm that the data supporting the findings of this study are available within the article and its supplementary materials.

## References

[1] Heintz E, Gerber-Grote A, Ghabri S, Hamers FF, Rupel VP, Slabe-Erker R, Davidson TWP (2016). 7 EUnetHTA Joint Action 2 Subgroup 3: Is there a European view on health economic evaluations? Results from a synopsis of methodological guidelines used in the EUnetHTA partner countries. Pharmacoeconomics.

[2] Goeree R, He J, O’Reilly D, Tarride J-E, Xie F, Lim M, Burke N (2011). Transferability of health technology assessments and economic evaluations: a systematic review of approaches for assessment and application. Clin Outcomes Res.

[3] McDonough CM, Tosteson ANA (2007). Measuring preferences for cost-utility analysis: how choice of method may influence decision-making. Pharmacoeconomics.

[4] Utility, YHEC - York Health Econ. Consort http://yhec.co.uk/glossary/utility/.

[5] Prieto L, Alonso J (2000). Exploring health preferences in sociodemographic and health related groups through the paired comparison of the items of the Nottingham Health Profile. J Epidemiol Community Health.

[6] Health outcomes in economic evaluation: the QALY and utilities. British Medical Bulletin.

[7] Drummond MF, Sculpher MJ, Claxton K, Stoddart GL, Torrance GW (2015). http://ebookcentral.proquest.com/lib/bristol/detail.action?docID=4605509.

[8] Oppe M, Rand-Hendriksen K, Shah K, Ramos-Goñi JM, Luo N (2016). EuroQol protocols for time trade-off valuation of health outcomes. Pharmacoeconomics.

[9] Dolan P (2008). Developing methods that really do value the “Q” in the QALY. Health Econ Policy Law.

[10] Russell LB, Gold MR, Siegel JE, Daniels N, Weinstein MC (1996). The role of cost-effectiveness analysis in health and medicine. Panel on Cost-Effectiveness in Health and Medicine. JAMA.

[11] (2013). 5 The reference case | Guide to the methods of technology appraisal 2013.

[12] Seixas BV (2017). Welfarism and extra-welfarism: a critical overview. Cad Saúde Pública.

[13] Brouwer WBF, Culyer AJ, van Exel NJA, Rutten FFH (2008). Welfarism vs. extra-welfarism. J Health Econ.

[14] Grutters JPC, Sculpher M, Briggs AH, Severens JL, Candel MJ, Stahl JE, De Ruysscher D, Boer A, Ramaekers BLT, Joore MA (2013). Acknowledging patient heterogeneity in economic evaluation. Pharmacoeconomics.

[15] Coast J (2019). Assessing capability in economic evaluation: a life course approach?. Eur J Health Econ.

[16] Sackett DL, Torrance GW (1978). The utility of different health states as perceived by the general public. J Chronic Dis.

[17] Papaioannou D, Sutton A, Carroll C, Booth A, Wong R (2010). Literature searching for social science systematic reviews: consideration of a range of search techniques. Health Inf Libr J.

[18] Schlosser RW, Wendt O, Bhavnani S, Nail-Chiwetalu B (2006). Use of information-seeking strategies for developing systematic reviews and engaging in evidence-based practice: the application of traditional and comprehensive pearl growing. A review Int J Lang Commun Disord.

[19] Mitchell PM, Roberts TE, Barton PM, Coast J (2017). Applications of the capability approach in the health field: a literature review. Soc Indic Res.

[20] M.V.H. Group (1994). The measurement and valuation of health. First Rep Main Surv.

[21] Dolan P (2000). Effect of age on health state valuations. J Health Serv Res Policy.

[22] Dolan P, Gudex C, Kind P, Williams A (1996). The time trade-off method: results from a general population study. Health Econ.

[23] Dolan P, Roberts J (2002). To what extent can we explain time trade-off values from other information about respondents?. Soc Sci Med.

[24] Document search - All Databases.

[25] JBI Critical Appraisal Tools.

[26] Badia X, Fernandez E, Segura A (1995). Influence of socio-demographic and health status variables on evaluation of health states in a Spanish population. Eur J Public Health.

[27] Badia X, Monserrat S, Roset M, Herdman M (1999). Feasibility, validity and test-retest reliability of scaling methods for health states: the visual analogue scale and the time trade-off. Qual Life Res.

[28] van Nooten F, Brouwer W (2004). The influence of subjective expectations about length and quality of life on time trade-off answers. Health Econ.

[29] Johnson JA, Luo N, Shaw JW, Kind P, Coons SJ (2005). Valuations of EQ-5D health states: are the United States and United Kingdom different?. Med Care.

[30] Essink-Bot M-L, Stuifbergen MC, Meerding W-J, Looman CWN, Bonsel GJ (2007). VOTE group, individual differences in the use of the response scale determine valuations of hypothetical health states: an empirical study. BMC Health Serv Res.

[31] Shaw JW, Johnson JA, Chen S, Levin JR, Coons SJ (2007). Racial/ethnic differences in preferences for the EQ-5D health states: results from the U.S. valuation study. J Clin Epidemiol.

[32] Jakubczyk M (2009). Impact of complementarity and heterogeneity on health related utility of life. Cent Eur J Econ Model Econom.

[33] van Nooten FE, Koolman X, Brouwer WBF (2009). The influence of subjective life expectancy on health state valuations using a 10 year TTO. Health Econ.

[34] Kharroubi SA, O’Hagan A, Brazier JE (2010). A comparison of United States and United Kingdom EQ-5D health states valuations using a nonparametric Bayesian method. Stat Med.

[35] Augestad LA, Rand-Hendriksen K, Stavem K, Kristiansen IS (2013). Time trade-off and attitudes toward euthanasia: implications of using “death” as an anchor in health state valuation. Qual Life Res.

[36] van Nooten FE, van Exel NJA, Koolman X, Brouwer WBF (2015). “Married with children” the influence of significant others in TTO exercises. Health Qual Life Outcomes.

[37] Krol M, Attema AE, van Exel J, Brouwer W (2016). Altruistic preferences in time tradeoff: consideration of effects on others in health state valuations. Med Decis Making.

[38] Santos M, Cintra MACT, Monteiro AL, Santos B, Gusmão-Filho F, Andrade MV, Noronha K, Cruz LN, Camey S, Tura B, Kind P (2016). Brazilian valuation of EQ-5D-3L health states: results from a saturation study. Med Decis Making.

[39] Jin X, Liu GG, Luo N, Li H, Guan H, Xie F (2016). Is bad living better than good death? Impact of demographic and cultural factors on health state preference. Qual Life Res.

[40] Sayah FA, Bansback N, Bryan S, Ohinmaa A, Poissant L, Pullenayegum E, Xie F, Johnson JA (2016). Determinants of time trade-off valuations for EQ-5D-5L health states: data from the Canadian EQ-5D-5L valuation study. Qual Life Res Int.

[41] van Nooten FE, Houghton K, van Exel J, van Agthoven M, Brouwer WBF, Stull DE (2017). A (Latent) class of their own: response patterns in trading off quantity and quality of life in time trade-off exercises. Value Health.

[42] Barry L, Hobbins A, Kelleher D, Shah K, Devlin N, Goni JMR, O’Neill C (2018). Euthanasia, religiosity and the valuation of health states: results from an Irish EQ5D5L valuation study and their implications for anchor values. Health Qual Life Outcomes.

[43] Kharroubi SA, Daher CA (2018). Modelling a preference-based index for EQ-5D using a non-parametric Bayesian method. Qual Life Res.

[44] Zhuo L, Xu L, Ye J, Sun S, Zhang Y, Burstrom K, Chen J (2018). Time trade-off value set for EQ-5D-3L based on a nationally representative chinese population survey, value health. J Int Soc Pharmacoeconomics Outcomes Res.

[45] Cubi-Molla P, Shah K, Garside J, Herdman M, Devlin N (2019). A note on the relationship between age and health-related quality of life assessment. Qual Life Res.

[46] Spencer A, Tomeny E, Mujica-Mota RE, Robinson A, Covey J, Pinto-Prades JL (2019). Do time trade-off values fully capture attitudes that are relevant to health-related choices?. Eur J Health Econ.

[47] Roudijk B, Donders ART, Stalmeier PFM (2019). Cultural values group, cultural values: can they explain differences in health utilities between countries?. Med Decis Mak Int J Soc Med Decis Mak.

[48] Santos M, Monteiro AL, Santos B (2020). Exploring the predictors of health valuations of EQ 5D 3L with a mixed-effects linear model. Expert Rev Pharmacoecon Outcomes Res.

[49] Hansen TM, Stavem K, Rand K (2022). Time trade-off with someone to live for: impact of having significant others on time trade-off valuations of hypothetical health states. Qual Life Res.

[50] Al Shabasy S, Al Sayah F, Abbassi M, Farid S (2022). Determinants of health preferences using data from the egyptian EQ-5D-5L valuation study. Patient.

[51] Page MJ, McKenzie JE, Bossuyt PM, Boutron I, Hoffmann TC, Mulrow CD, Shamseer L, Tetzlaff JM, Akl EA, Brennan SE, Chou R (2021). The PRISMA 2020 statement: an updated guideline for reporting systematic reviews. Syst Rev.

[52] Kharroubi S, Brazier JE, O’Hagan A (2007). Modelling covariates for the SF-6D standard gamble health state preference data using a nonparametric Bayesian method. Soc Sci Med.

[53] Kharroubi SA, McCabe C (2008). Modeling HUI 2 health state preference data using a nonparametric Bayesian method. Med Decis Making.

[54] Kharroubi SA, Brazier JE, McGhee S (2014). A comparison of Hong Kong and United Kingdom SF-6D health states valuations using a nonparametric Bayesian method. Value Health.

[55] Wittenberg E, Halpern E, Divi N, Prosser LA, Araki SS, Weeks JC (2006). The effect of age, race and gender on preference scores for hypothetical health states. Qual Life Res.

[56] Grewal I, Lewis J, Flynn T, Brown J, Bond J, Coast J (2006). Developing attributes for a generic quality of life measure for older people: preferences or capabilities?. Soc Sci Med.

[57] Hofman CS, Makai P, Boter H, Buurman BM, de Craen AJ, Olde Rikkert MGM, Donders R, Melis RJF (2015). The influence of age on health valuations: the older olds prefer functional independence while the younger olds prefer less morbidity. Clin Interv Aging.

[58] Robinson A, Dolan P, Williams A (1997). Valuing health status using VAS and TTO: what lies behind the numbers?. Soc Sci Med.

[59] Sculpher M, Gafni A (2001). Recognizing diversity in public preferences: the use of preference sub-groups in cost-effectiveness analysis. Health Econ.

