# Supplementary material for: Preferences for health-related quality of life: do they vary by age? A systematic literature review on the EQ-5D measure
Source: Eur J Health Econ. 2025 Mar 25;26(7):1275–91. doi: 10.1007/s10198-025-01766-7 (PMC7617589; doi:10.1007/s10198-025-01766-7)
Supplement: Supplementary file 1 — Supplementary file1 (DOCX 15 KB) [file 10198_2025_1766_MOESM1_ESM.docx]

**Appendix 1: Databases search strategy and PICO framework.**

The following key terms and Medical Subject Headings (MeSH) were searched across the PsycINFO, PubMed, Medline, and Embase databases using the OVID platform, covering the period from 1994 to July 2023, on 12/07/23. A subject librarian from Bristol Medical School reviewed the search strategy.

| **Population** | **AND** | **Intervention** | **AND** | **Comparator** | **AND** | **Outcome** | **AND** | **Methods** | **Search Result** |
| --- | --- | --- | --- | --- | --- | --- | --- | --- | --- |
| “general population” .tw. (359213)  OR “older people” .tw. (100090)  OR “older adult*” .tw. (331457)  OR elderly.tw. (762179) |  | EQ-5D*.tw. (37,838)  OR EQ5D*.tw. (4016)  OR EuroQol.tw. (21035) |  | exp Aged/ (7101273)  OR age factors/ (991554)  OR age.tw. (7894427)  OR health status/ or geriatric assessment/ (295451) |  | “preference valuation” .tw. (28)  OR utilit*.tw. (712653)  OR valu*.tw. (712653) |  | “standard gamble” .mp. (2364)  OR “time trade-off” .mp. (3937)  OR “visual analogue scale” .mp. (98865)  OR “discrete choice experiment*“.mp. (8000)  OR ranking.mp. (83050)  OR “Surveys and Questionnaires”/ec, mt [Economics, Methods] (198) | Total results: (630)  Results after removing duplicates and language filter: (413) |

**Commands Key:**

| * | Allows to search for all terms that begin with the same word stem. |
| --- | --- |
| .tw. | Retrieves records that contain the terms in the title, abstract, and keyword fields. |
| exp | Retrieves all references containing the subject heading, plus any more specific subject headings. |
| / | Denotes a MeSH (Medical Subject Headings) heading. |
| .mp. | Searches in several fields, including title, abstract, original title, name of substance word, subject heading word, protocol supplementary concept, and rare disease supplementary concept. |
| ec | Economics [Subheading] |
| mt | Methods [Subheading] |
