# Supplementary material for: Preferences for health-related quality of life: do they vary by age? A systematic literature review on the EQ-5D measure
Source: Eur J Health Econ. 2025 Mar 25;26(7):1275–91. doi: 10.1007/s10198-025-01766-7 (PMC7617589; doi:10.1007/s10198-025-01766-7)
Supplement: Supplementary file 2 — Supplementary file2 (DOCX 36 KB) [file 10198_2025_1766_MOESM2_ESM.docx]

**Appendix 2: Risk of bias and quality assessment using the Joanna Briggs Institute critical appraisal tool for cross-sectional studies.**

| Study ID | Dolan et al., 1996 (22) | Dolan, P., 2000 (21) | Dolan, P. et al., 2002 (23) | Kharroubi, S.A., et al., 2018 (43) | Cubi-Molla, P. et al., 2019 (45) | Barry, L. et al., 2018, (42) | Spencer et al., 2019 (46) | Shaw et al., 2007 (31) | Johnson, J.A. et al., 2005 (29) | Kharroubi, S.A., et al., 2010 (34) | Santos, M., et al., 2016 (38) | Santos, M. et al., 2020 (48) | Sayah, F.A. et al., 2016 (40) | Al Shabasy, S. et al., 2022 (50) | Jakubczyk, M., 2009 (32) | Jin et al. (2016) (39) | Zhuo, L. et al., 2018 (44) | Hansen, T.M., et al., 2022 (49) | Augestad et al., 2013 (35) | Badia, X., 1995 (26) | Badia et al. 1999 (27) | Van Nooten et al., 2009 (33) | Bot et al., 2007 (30) | Van Nooten, F.E., et al., 2015 (36) | Nooten et al., 2017 (41) | Roudijk B et al., 2019 (47) | Krol, M. et al., 2016 (37) | Van Nooten et al., 2004 (28) |
| --- | --- | --- | --- | --- | --- | --- | --- | --- | --- | --- | --- | --- | --- | --- | --- | --- | --- | --- | --- | --- | --- | --- | --- | --- | --- | --- | --- | --- |
| Were the criteria for inclusion in the sample clearly defined? | Y | Y | Y | Y | Y | Y | Y | Y | Y | Y | Y | Y | Y | Y | Y | Y | Y | Y | Y | Y | Y | Y | N | Y | Y | Y | Y | N |
| Were the study subjects and the setting described in detail? | Y | Y | Y | Y | Y | Y | Y | Y | Y | Y | Y | Y | Y | Y | Y | Y | Y | Y | Y | Y | Y | Y | Y | Y | Y | Y | UC | N |
| Was the exposure measured in a valid and reliable way? | Y | Y | Y | Y | Y | Y | UC | Y | Y | Y | Y | Y | Y | Y | N | Y | Y | Y | Y | Y | Y | Y | Y | Y | Y | Y | Y | Y |
| Were objective, standard criteria used for measurement of the condition? ^a^ | Not applicable | | | | | | | | | | | | | | | | | | | | | | | | | | | |
| Were confounding factors identified? | Y | N | Y | N | Y | Y | Y | Y | Y | Y | Y | Y | Y | Y | Y | Y | Y | Y | Y | Y | Y | Y | Y | Y | Y | UC | Y | Y |
| Were strategies to deal with confounding factors stated? | Y | N | Y | N | Y | Y | Y | Y | Y | Y | Y | Y | Y | Y | Y | Y | Y | Y | Y | Y | Y | Y | Y | Y | Y | UC | Y | Y |
| Were the outcomes measured in a valid and reliable way? | Y | Y | Y | Y | Y | Y | Y | Y | Y | Y | Y | Y | Y | Y | Y | Y | UC | Y | Y | Y | Y | Y | Y | Y | Y | N | Y | Y |
| Was appropriate statistical analysis used? | Y | Y | Y | Y | Y | Y | Y | Y | Y | Y | Y | Y | Y | Y | Y | Y | Y | Y | Y | Y | Y | Y | Y | Y | Y | Y | Y | Y |

^a^ This item assesses whether participants were chosen based on a diagnosis or definition. For our included studies, this was considered not applicable

Y: Yes

N: No

UN: Unclear
