# Supplementary material for: Preferences for health-related quality of life: do they vary by age? A systematic literature review on the EQ-5D measure
Source: Eur J Health Econ. 2025 Mar 25;26(7):1275–91. doi: 10.1007/s10198-025-01766-7 (PMC7617589; doi:10.1007/s10198-025-01766-7)
Supplement: Supplementary file 3 — Supplementary file3 (DOCX 47 KB) [file 10198_2025_1766_MOESM3_ESM.docx]

**Appendix 3:** **Statistical results in included Studies.**

| **Study ID** | **Statistical test** | **Dependent variables** | **Independent variables** | **Statistical results** |
| --- | --- | --- | --- | --- |
| Dolan et al., 1996,UK (22) | Ordinary Least Squares regression (OLS) | TTO values | Sex, age, age^2^, education, social class, marital status, and health status defined as problem in any dimension of the EQ-5D | Coefficient estimates and (t-statistics):  *Mild states:*  Age: 0.008 (5.83)  Age^2^: -0.00010 (-7.11)  *Moderate states:*  Age: 0.010 (4.81)  Age^2^: -0.00013 (-6.45)  *Severe states:*  Age: 0.008 (5.400)  Age^2^: - 0.00012 (-8.100) |
| Dolan, P., 2000, UK (21) | Generalized least-squares regression | 1-TTO values | Two dummy variables for each dimension: one to represent the move between levels within health states and one to represent the move from level 2 to level 3 | Decrements for health state utility value by age:  *Constant representing any move away from full health*  18-59: 0.076  ≥ 60: 0.090, ≥60 Modified: 0.090  *Decrement when any dimension is at level 3*  18-59: 0.236  ≥ 60: 0.346, ≥60 Modified: 0.2555  *Some problems washing/dressing self*  18-59: 0.083  ≥ 60: 0.150, ≥60 Modified: 0.099  *Unable to wash/dress self*  18-59: 0.199  ≥60: 0.248, ≥60 Modified: 0.201 |
| Dolan, P. et al., 2002, UK (23) | Random effect model | TTO values | VAS valuation, age, sex, marital status, own health, interaction between age and Visual Analogue Scale (VAS), and the interaction between sex and VAS | Coefficient estimates and (t-statistics):  Age: 0.009 (0.002)  Age^2^: -0.0001 (0.00002) |
| Kharroubi, S.A., et al., 2018, UK (43) | Non-parametric bayesian methods | TTO values | Age, age^2^, sex | NR |
| Spencer et al., 2019, UK (46) | Regression analysis | TTO values | Age, age^2^, marital status, age left school, health states valued, and attitude towards quality and length of life | Coefficient estimates, (SE) and P value:  Age: 0.016, (0.003), 0.000  Age^2^:-0.0001, (0.00). 0.000 |
| Barry, L. et al., 2018, UK (42) | Bivariate probit model | The probability of respondents assigning WTD values to a health state. | Age, gender, marital status, education, self-reported health (VAS), experience of a serious illness, having dependents under 18, and attitude towards euthanasia | Coefficient estimates, (SE), P value:  Age  *(Reference group: 18–35)*  (36–45): 0.304 (0.207), P >0.05  (46–60): 0.471 (0.228), P <0.05  (61+): 1.16 (0.274), P <0.001 |
| Cubi-Molla, P. et al.,  2019, UK (45) | OLS, ANOVA and Pairwise comparison | TTO values | Sex, self-reported health state, education, and personal experience with serious illness | Average TTO value by age group:  The reference group (68+)  **12211:** 68+ (0.6279), 18-27 (0.7356), 28-37 (0.8006**), 38-47 (0.8119**), 48-57 (0.8341**), 58-67 (0.8054**).  **23313**: 68+ (-0.2329), 18-27 (-0.0535), 28-37 (0.0463**), 38-47 (-0.0807),  48-57 (-0.0808), 58-67 (-0.0396).  **32211:** 68+ (-0.0806), 18-27 (0.1634), 28-37 (0.2266**), 38-47 (0.2876**),  48-57 (0.2850**), 58-67 (0.0009).  **32223**: 68+ (-0.3810), 18-27 (-0.2076), 28-37 (-0.1182), 38-47 (-0.0836), 48-57 (0.0087**), 58-67 (-0.2418).  **32232**: 68+ (-0.4059), 18-27 (-0.1765), 28-37 (-0.2016), 38-47 (-0.1344), 48-57 (-0.0905**), 58-67 (-0.2857).  **32331**: 68+ (-0.4718), 18-27 (-0.3081), 28-37 (-0.1753), 38-47 (-0.1677**), 48-57 (-0.1970), 58-67 (-0.3688).  **33212:** 68+ (-0.2490), 18-27 (0.0484), 28-37 (0.0644**), 38-47 (0.0864**), 48-57 (0.0549), 58-67 (-0.1397).  **Indicates a statistically significant difference at the 99% level. |
| Shaw et al., 2007, US (31) | Multiple linear regression | TTO values | Age, age^2^, region, ethnicity, sex, education, marital status, self-rated health status, health insurance, belief in life after death, experience caring for the ill, chronic conditions, and household income | Coefficient estimates and (t-statistics):  Age/10: 0.070 (4.14)  Age^2^ /1000: -0.089 (-4.81) |
| Johnson, J.A. et al., 2005, US (29) | Linear regression Analyses. Random-effects model. | TTO values | Country (US/UK), age, age^2^, sex, and dummies for the interaction of US with other variables | Coefficient estimates and (P value):  US: 0.095 (<0.0001)  Age: 0.011 (<0.0001)  Age^2^: -0.0001 (<0.0001)  US X Age: -0.001 (<0.7)  US X Age^2^: <0.0001 (<0.6) |
| Kharroubi, S.A., et al., 2010, UK (34) | Non-parametric bayesian methods | TTO values | Country (US/UK), age, age^2^, sex, and dummies for the interaction of the US with other variables | Estimated coefficients:  Age: -0.014  US X Age: 0.004  P values were not reported |
| Santos, M., et al., 2016, Brazil (38) | OLS, robust linear model, and a mixed-effect model. | TTO values | Dummy variables for each health dimension severity level | NR |
| Santos, M. et al., 2020, Brazil (48) | Mixed-effect linear model | TTO values | Age, marital state, belief in god or the afterlife, sex, education, ownership of goods, having children, unemployment, health insurance, having a recently deceased friend, smoking, number of chronic diseases, and happiness level | Coefficient estimates (P value) and 95% confidence interval (CI)  Age: 0.0019, (0.0000), (0.001, 0.002) |
| Sayah, F.A. et al., 2016, Canada (40) | Univariate linear models, multivariable random-effects linear regression. | TTO values | VAS score, age, sex, marital status, education, employment, household income, ethnicity, country of birth, and study site | Coefficient estimates, standard error (SE), (P value):  *The reference group (18-28)*  *Univariant analysis:*  Age: (40-59)  -0.003, SE = 0.023, P = 0.91  Age: (60-89)  -0.067, SE = 0.025, P = 0.007  *Multivariate analysis:*  Age: (40-59)  -0.029, SE = 0.023, P = 0.2  Age: (60-89)  -0.077, SE = 0.027, P= 0.004 |
| Al Shabasy, S. et al., 2022, Egypt (50) | Univariate linear models, multivariable random-effects linear regression. | TTO values | Age, sex, education, region, religion, marital status, number of  people in the household, employment, health insurance, number of self-reported chronic conditions, preferences for quality or quantity of life, and experience of serious illness | Coefficient estimates, SE, (P value):  *The reference group (18-30)*  *Univariant analysis:*  Age: (31-45)  -0.024, SE = 0.011, P = 0.024  Age: (46-60)  -0.024, SE = 0.012, P = 0.051  Age: 60+  -0.212, SE = 0.029, P < 0.001  *Multivariate analysis:*  Age: (31-45)  -0.044, SE = 0.015, P = 0.004  Age: (46-60)  -0.026, SE = 0.018, P = 0.152  Age: 60+  -0.187, SE = 0.033, P < 0.001 |
| Jakubczyk, M., 2009, Poland (32) | Random effect model | 1-TTO values | Sex, age, respondents coming from Warsaw, respondents from the countryside, education, strong belief in life after death, and self-health | Coefficient estimates, SE, (P value):  Age: -0.0051, SE=0.001, P = 0.000 |
| Jin et al. 2016, China (39) | Generalised linear regression and random effect model. | TTO = 1  TTO ≥ 0  TTTO= -1  TTO range | Age, gender, education, employment, residence, health insurance, experience with serious diseases, responses to whether bad living is better than good death, and afterlife questions | No significant relationship was found between age and all four indicators  P value > 0.05 |
| Hansen, T.M., et al., 2022, Norway (49) | Linear regression and random effect model. | 1-TTO values and  number of years traded off | Children, partner, children or partner, significant others, age, gender, and education | Coefficient estimates, SE, and P value:  Age Quartile 1:  0.176, (0.104), P <0.1  Age Quartile 2:  0.056, (0.104), P >0.1  Age Quartile 3:  0.634 ( 0.200), P <0.01  Age Quartile 4:  0.385 (0.148), P <0.001 |
| Augestad et al., 2013, Norway (35) | Linear regression | TTO, LT-TTO and VAS scores.  Number of health states considered WTD | Age, gender, education, and attitude toward euthanasia | Coefficient estimates, SE, and P value:  *Mean utility scores:*  TTO:  Age: 0.002, (0.001), P = 0.043  LT-TTO:  Age: -0.001, (0.001), P = 0.254  VASir: (Rescaled VAS values by individual values for state 11111 and ‘death’)  Age: -0.001, (0.001), P= 0.134  VASmr: (Rescaled VAS values by mean values for state 11111 and ‘death’)  Age: 0.001, (0.00), P = 0.037  *The number of states valued WTD:*  TTO:  Age: -0.002, (0.003), P= 0.451  LT-TTO:  Age: 0.008, (0.003), P = 0.004  VASir:  Age: -0.01 (0.001), P = 0.000  VASmr:  Age: -0.004 (0.001), P<0.001 |
| Badia, X., 1995, Spain (26) | One-way ANOVA, multiple linear regression and chi-squared test | VAS values | Sex, age, occupation, education, task difficulty, and self-health | NR |
| Badia et al. 1999, Spain (27) | Multiple regression analysis and chi-square test | TTO and VAS values | Age, gender, education, presence of health problems, self-rated health, and experience of illness | NR |
| Van Nooten et al., 2009, Netherlands (33) | Generalised negative binomial model and a probit model | WTT years  And the number of years traded off | Age, gender, quality of life, education, the difference between current age and the expected age of death according to the respondents (SLE), and a variable indicating whether the expected age of death was less than 10 years | Coefficient estimates and 95% CI  *Probit model (Willingness to trade off) :*  Age: -0.00162, (−0.003, 0.0005)  SLE: -0.00187, (−0.003, −0.00002)  *General binomial regression: (number of years traded off)*  Age: -0.0108 (−0.019, −0.002)  SLE: -0.0091 (−0.016, −0.0014) |
| Bot et al., 2007, Netherlands (30) | One-way ANOVA, G-theory study, Principal Component Analysis and Ordination Based Cluster Analysis | TTO, TTO-LE and VAS values | Health state, method, age group, respondent [age group], first-order interactions health state * method, health state * age group, health state * respondent [age group], method * age group, and method * respondent [age group] | G-study on all elicitation methods collectively: (% of variance in utility values explained by each facet)  Age group: 0.2%  Health state x Age group: 0.0%  Method x Age group: 0.0% |
| Van Nooten,, et al., 2015, Netherlands (36) | Regression model | Number of years traded off | Gender, age, highest education, marital status, children, number of children, age  Of the youngest child, health (VAS; chronic illness; serious illness; weight), and expectations (subjective life expectancy and quality of life at the ages of 60, 70, 80 and 90) | Coefficient Estimates and 95% CI:  *Model 1 (excluding the effect of living with a partner):*  Age: −0.044, (−0.057, −0.032)  *Model 2 (including the effect of living with a partner)*  Age: −0.044, (−0.053, −0.036)  *Model 3 (including living with a partner and children)*  Age: −0.04, (−0.049, −0.032) |
| Nooten et al., 2017, Netherlands (41) | Latent class analysis | Number of years traded off | Age, sex, education, marital status, having children, quality of life (based on EQ-5D VAS), subjective life expectancy, and the stated preference for quality versus quantity of life | Coefficient estimates, (SE) and P value  *The relationship between age and the likelihood of being in a specific trader group:*  *The high trader is the reference class:*  Age:  Low traders  0.065, ( 0.014), P < 0.001  Medium-low traders  0.039, (0.015), P = 0.010  Medium-high traders  0.032, (0.012) P = 0.010 |
| Roudijk B et al., 2019, Netherlands (47) | Two-Level Mixed-Effects Models and Intraclass Correlation Coefficient | TTO differences between mild and severe states | Age, sex, education, and EQ-5D self-description | Correlation coefficient, (96% CI) or P value:  *Country-level analysis:*  Age:  -0.119, (-0.447, 0.274)  *Analysis Across 27 Countries:*  Age:  0.004, (P > 0.05) |
| Zhuo, L. et al., 2018, China (44) | Multiple linear regression | TTO values | Sex, age group, region, education, employment, marital status, and economic status | Coefficient estimates and P values:  Age:  *(15-24) reference group*  (25-34): 0.026, 0.000  (35-44): 0.018, 0.016  (45-54): 0.014, 0.051  (55-64): 0.032, 0.000  (65-74): 0.039, 0.000  (75-97): 0.032, 0.000 |
| Krol, M. et al., 2016, Netherlands (37) | Generalized Estimating Equations | TTO values | Age, gender, self-assessed health, children, religion, partner, education, employment, studying, and thoughts of: loved ones, social role functioning, being a burden and being missed | Coefficient estimates, (SE) and P value:  Age:  0.001, (0.0008), 0.31  Thought of being a burden:  -0.076, (0.0233), 0.001  Thought of being missed:  0.114, (0.024), <0.001 |
| Van Nooten et al., 2004, Netherlands (28) | OLS | TTO values and number of years traded off | Gender, age, education, quality of life now, at 60, at 70, at 80, at 90, and expected age of death | Coefficient estimates, (SD), and P values  *Mean utility values:*  Age:  11211: 0.00 P <0.1  22222: -0.000 P > 0.1  11232: -0.001 P > 0.1  Expected age of death:  11211:0.00 P > 0.1  22222: 0.005 P < 0.001  11232: 0.005 P < 0.001  *Number of years traded off:*  11211:  Life expectancy (<80): 0.69, (>80): 0.34  22222:  Life expectancy (<80): 6.1, (>80): 2.77  11232:  Life expectancy (<80): 9.27, (>80): 5.77  All differences were significant, P <0.01 |
